# Correction: The Antsy Social Network: Determinants of Nest Structure and Arrangement in Asian Weaver Ants

**DOI:** 10.1371/journal.pone.0159284

**Published:** 2016-07-08

**Authors:** Kadambari Devarajan

## Notice of Republication

This article was republished on June 29, 2016, to correct the order of images for Figs 2–9, which were published out of order. The author apologizes for the errors. Please download this article again to view the correct version. The originally published, uncorrected article and the republished, corrected article are provided here for reference.

## Supporting Information

S1 File Originally published, uncorrected article. (PDF)

S2 File Republished, corrected article. (PDF)
