# Supplementary material for: Correction: The Antsy Social Network: Determinants of Nest Structure and Arrangement in Asian Weaver Ants
Source: PLoS One. 2016 Jul 8;11(7):e0159284. doi: 10.1371/journal.pone.0159284 (PMC4938126; doi:10.1371/journal.pone.0159284)
Supplement: S1 File — (PDF) [file pone.0159284.s001.PDF]

PLOS ONE

RESEARCH ARTICLE

The Antsy Social Network: Determinants of Nest Structure and Arrangement in Asian Weaver Ants

Kadambari Devarajan*

Post Graduate Programme in Wildlife Biology and Conservation, Wildlife Conservation Society - India Program, National Centre for Biological Sciences, Bengaluru, India

* kadambari.devarajan@gmail.com

CrossMark
click for updates

OPEN ACCESS

Citation: Devarajan K (2016) The Antsy Social Network: Determinants of Nest Structure and Arrangement in Asian Weaver Ants. PLoS ONE 11 (6): e0156681. doi:10.1371/journal.pone.0156681

Editor: Fabio S. Nascimento, Universidade de São Paulo, Faculdade de Filosofia Ciências e Letras de Ribeirão Preto, BRAZIL

Received: July 1, 2014

Accepted: May 18, 2016

Published: June 7, 2016

Copyright: © 2016 Kadambari Devarajan. This is an open access article distributed under the terms of the Creative Commons Attribution License, which permits unrestricted use, distribution, and reproduction in any medium, provided the original author and source are credited.

Data Availability Statement: All data files are available from the Figshare database (DOI: http://dx.doi.org/10.6084/m9.figshare.1380112). Devarajan, Kadambari (2015): Weaver Ant Nest Data. figshare. http://dx.doi.org/10.6084/m9.figshare.1380112.

Funding: Funding was provided by the National Centre for Biological Sciences (URL: http://ncbs.res.in/). The funders had no role in study design, data collection and analysis, decision to publish, or preparation of the manuscript.

Competing Interests: The author has declared that no competing interests exist.

Abstract

Asian weaver ants (*Oecophylla smaragdina*) are arboreal ants that are known to form mutualistic complexes with their host trees. They are eusocial ants that build elaborate nests in the canopy in tropical areas. A colony comprises of multiple nests, usually on multiple trees, and the boundaries of the colony may be difficult to identify. However, they provide the ideal model for studying group living in invertebrates since there are a definite number of nests for a given substrate, the tree. Here, we briefly examine the structure of the nests and the processes involved in the construction and maintenance of these nests. We have described the spatial arrangement of weaver ant nests on trees in two distinct tropical clusters, a few hundred kilometres apart in India. Measurements were made for 13 trees with a total of 71 nests in the two field sites. We have considered a host of biotic and abiotic factors that may be crucial in determining the location of the nesting site by Asian weaver ants. Our results indicate that tree characteristics and architecture followed by leaf features help determine nest location in Asian weaver ants. While environmental factors may not be as influential to nest arrangement, they seem to be important determinants of nest structure. The parameters that may be considered in establishing the nests could be crucial in picking the evolutionary drivers for colonial living in social organisms.

Introduction

Weaver ants are eusocial insects, belonging to the family Formicidae and order Hymenoptera, that have a unique nest-building behavior. A number of ant species, belonging to different genera such as *Polyrhachis* and *Dendromyrmex*, use larval silk in the construction of nests. The term "weaver ants" typically refers to two closely-related extant species, both belonging to the genus *Oecophylla*. African weaver ants (*Oecophylla longinoda*) are found in sub-Saharan Africa, while Asian weaver ants (*Oecophylla smaragdina*) are found in Australasia. A number of studies have been made on weaver ants from the time they were first described [1–6] with a focus on eusociality [2–6], interactions with plants [7, 8], and on their use as biological pest control agents [9–13].

Even though the Asian weaver ant, *Oecophylla smaragdina*, is widely distributed across the Indian subcontinent and south-east Asia extending up to Australia, very little research has been done on them, especially in India. However, since they are an excellent model for studying eusociality, there have also been studies on their chemical ecology [14–16], population and colony structure [4, 6], breeding system [6], and communication [15, 16]. Some studies on weaver ants have focused on their evolution [3, 17], behavior [18, 19], and ecology [7, 19, 20]. Out of the studies pertaining to the nests of weaver ants [1, 2, 21], some have tried to understand nest construction and maintenance, but not habitat use or nest arrangement.

Weaver ants build elaborate nests, varying in size, by weaving together leaves of the host plant using the silk produced by their larvae. For this reason, the aspect of sociality in weaver ants is considered amongst the most refined within hymenopterans [6]. These eusocial ants also interact with other organisms in a variety of ways. In myrmecophytic systems, pugnacious ants protect their host plant in return for resources such as food (typically extra-floral nectar) and shelter (known as domatia) [22–28]. These have been pivotal in our current understanding of mutualisms and remain one of the best models to investigate the dynamics of mutualistic interactions. While such protection by the ants in return for rewards is thought to be common to ant-plant mutualistic complexes, recent research indicates that the plants could also be deterring the ants from other resources (such as nutrients) or other mutualistic interactions the plant may have [26–28]. Such complexes need to be studied well to understand interactions between organisms better.

Here, we look at one such system involving Asian weaver ants in an attempt to understand their interactions with the host plant and the environment. This interaction was studied through investigations of nest structure, construction, and arrangement. The aim of this study was twofold—1) to understand the structure of weaver ant nests as well as the processes involved in their construction, and 2) to determine if the location of each weaver ant nest is influenced by one or more biotic and abiotic parameters. The focus here is on the specific variables that could affect the habitat use and local distribution of *O. smaragdina*. In particular, we ask if 1) tree characteristics (tree structure and leaf size), 2) environmental variables (temperature, luminosity, wind speed, and wind direction), and 3) nest characteristics (nest size and relative position), could help determine the nest architecture and arrangement in Asian weaver ants.

We hypothesise that tree characteristics and leaf features could be crucial to the selection of nesting sites by Asian weaver ants. Fruit trees could be involved in mutualistic complexes with weaver ants, especially in tropical areas, with the latter utilizing plant resources in return for defense against invertebrate or vertebrate visitors. We also predict that environmental factors could be determinants of nest structure while tree features are crucial predictors of nest architecture in Asian weaver ants.

Materials and Methods

Ethics Statement

The study was conducted outside protected areas in Karnataka and Tamil Nadu and research permits were not required for the study. Since the methods used were non-invasive and protected species were not sampled, animal ethics committee approval was not required. No human or vertebrate animal subjects and/or tissue were involved in this study.

Study Area

Asian weaver ant nests were surveyed across two distinct urban locations in southern India that are approximately 350 km apart. Nest surveys were conducted at various locations inside

the University of Agricultural Sciences (UAS) campus in the city of Bangalore as well as the Indian Institute of Technology Madras (IITM) (12°59′29”N, 80°14′1”E) campus in Chennai. A majority of the sampling inside the UAS campus was done within the National Center for Biological Sciences (NCBS) complex (13°4′56”N, 77°34′35″E), where four trees and 31 nests were surveyed. Extensive observational studies for nest structure and construction were made on some of these nests. At IITM, nine trees and 40 nests were surveyed across the campus. In total, 13 trees and 71 nests were surveyed. Both locations are areas of high-intensity use by humans as well as other vertebrates. However, despite being urban locations, both sites have large swathes of contiguous, undisturbed native vegetation.

For each nest, 1) its distance from the ground, 2) the distance from the trunk, 3) the distance to nearest neighbours, 4) the nest size in number of leaves, 5) the general size (“large”, “medium”, or “small” based on the volume), and use (“yes” if in use and “no” if abandoned), and 6) the angle and position with reference to the compass North, were recorded. In addition, environmental variables such as 1) the ambient temperature outside the nest, 2) wind intensity, 3) wind direction, 4) luminosity, and 5) humidity, for each nest were noted. Such abiotic factors tend to vary depending on the geography of the location, season, time of day, and a number of other conditions. This issue of variability was addressed by ensuring that all data was collected within the same season and at the same time of day for each site. Covariates related to the tree such as 1) tree species, 2) canopy size, 3) trunk height, 4) tree height, 5) leaf size (length and breadth at the widest ends), and 6) leaf shape, were noted, as well. The trunk was defined to be the portion of the tree until the point of first branching. Girth at breast height (GBH) was not considered since these are canopy ants and the canopy size with tree height and trunk height seemed to be more relevant factors. The latitude and longitude for each tree and nest were recorded using a GPS. Measurements were made using a Beetech ultrasonic distance meter, measuring tape, ladder, measured collapsible pipes, compass, and protracter. Enviromental variables were measured using a GPS, Kestrel weathermeter, luxmeter, and sensitive thermometer (at NCBS)/resistance thermometer (at IITM), respectively. Additionally, photographs were taken for each nest wherever possible and of the positions on the tree.

To study the nest structure and construction, scissors were used to make incisions, forceps of varying sizes and lengths to pry and observe, measuring tape for measuring lengths of the incisions made, and a clock for timing the duration taken for repair. We studied the process of nest construction by observing 1) the stitching of nests when just starting, 2) the mechanisms of extending nests that are already built, and 3) the process of maintenance. Maintenance was observed by damaging a few nests and noting the response. This was done only on weaver ant nests in the NCBS campus.

Study Species

The Asian weaver ant (*Oecophylla smaragdina*) is a dominant arboreal ant that is widely distributed across tropical Australasia [4, 5] (Fig 1). These ants build complex nests in the canopy that are frequently spread across multiple trees to form a single colony with hundreds of ants in each nest and thousands of ants in a colony. The *O. smaragdina* workers weave leaves of the host plant with silk from their larvae [1, 4, 5]. As with other social organisms that live in groups or colonies, it can be difficult to determine the exact boundaries of a colony of Asian weaver ants. While weaver ants have a wide distribution (from south Asia to Australia), most studies on weaver ants have historically been limited to sub-Saharan Africa (*Oecophylla longinoda*), neotropical America (*Camponotus senex*), and Australasia (*Oecophylla smaragdina*).

Weaver ant colonies could comprise of up to half a million ants that are housed in nests which could be spread across dozens of trees. The basic family structure for each colony

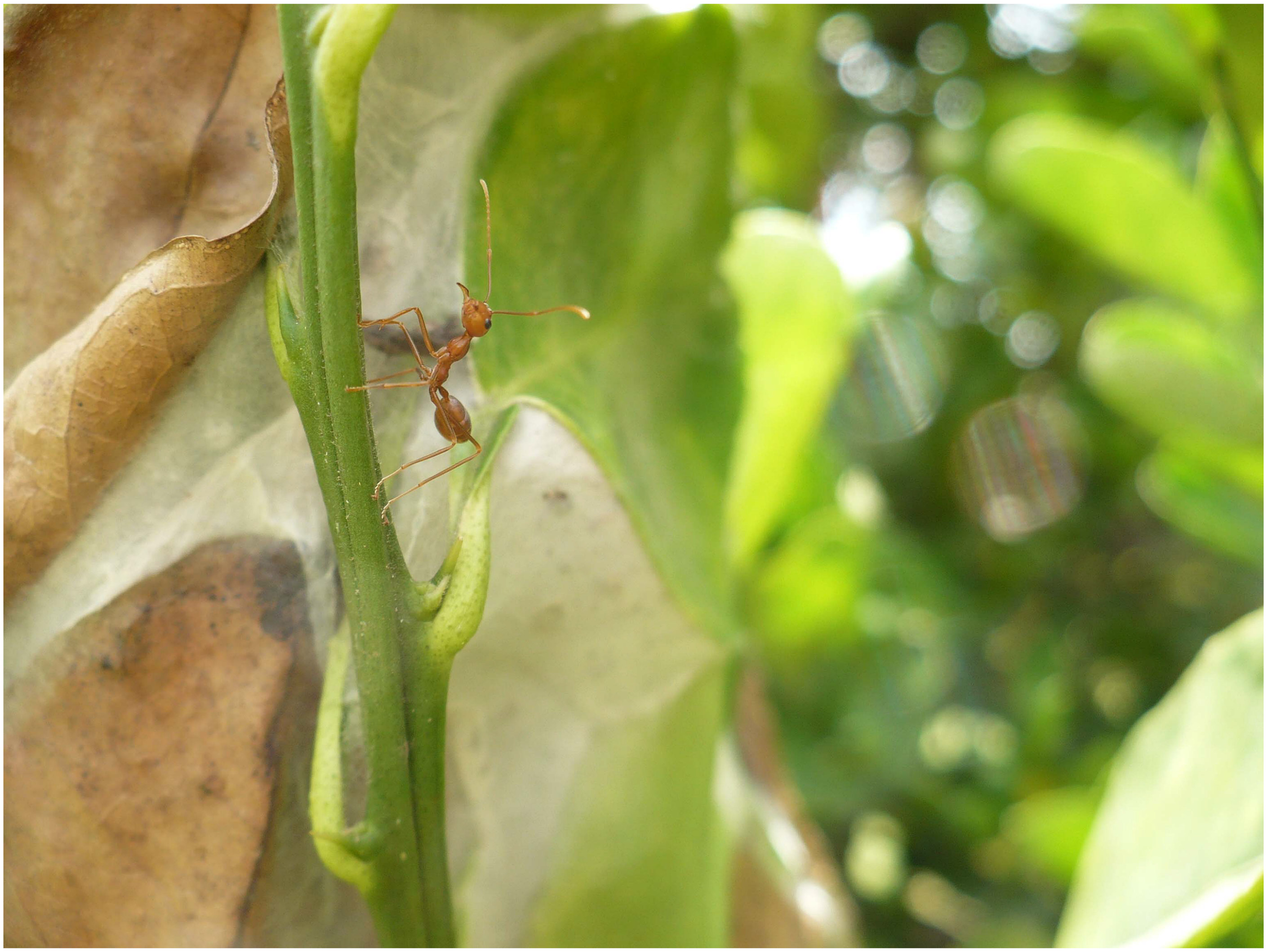

Fig 1. ***Oecophylla smaragdina*** **with the nest in the background.**

doi:10.1371/journal.pone.0156681.g001

comprises of a single mated queen and her worker offspring numbering in the hundred thousands, at times. This means that two colonies can be distinguished in one of two ways: 1) if two queens are found, which is very difficult given that each colony has multiple nests and that the number of nests in a colony is highly variable, and 2) through behavioral experiments on ants that are thought to be from different colonies.

In addition to nest building, the workers are also involved with foraging, exploring, and patrolling the area surrounding the nest, as well as caring for the queen and maintenance of the nest. A large fraction of a nest, and consequently a colony, is immobile at a given time. This could be a measure to conserve energy since the construction and maintenance of the nest is a huge investment of time and energy by the weaver ants.

Analysis

In order to identify the primary factors that influence the local arrangment of weaver ant nests on the trees, two statistical techniques were used, namely: Principal Component Analysis

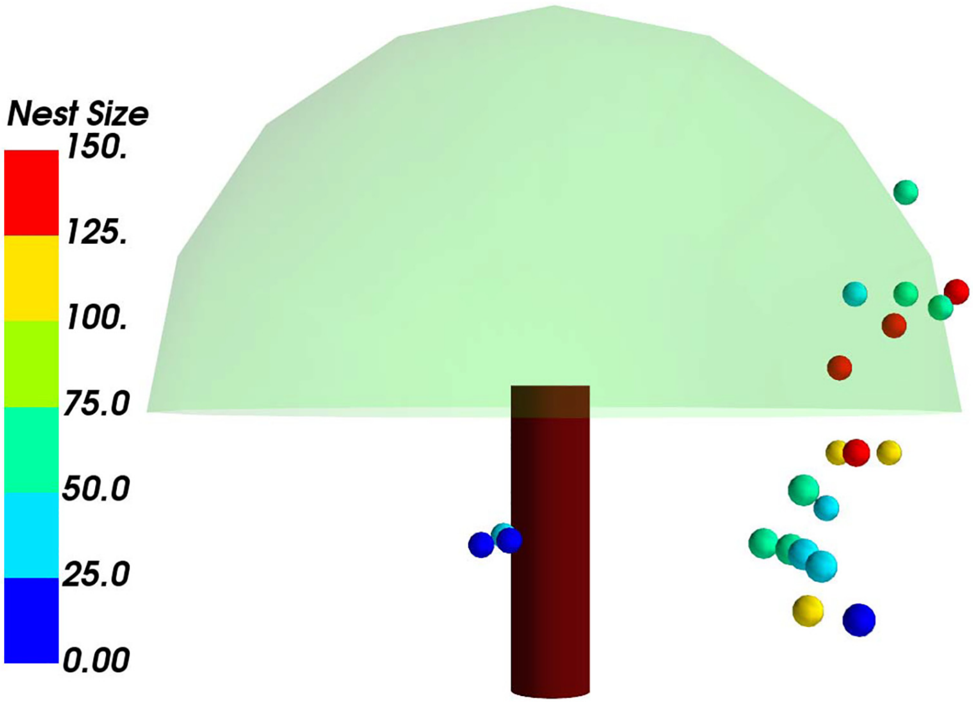

Fig 2. Scree plot showing the principal components that explain the most variation. Here, the first five components explain about 90% of the variation. The first two components account for 48% and 18% of the variation respectively, leading to a cumulative of 66%.

doi:10.1371/journal.pone.0156681.g002

(PCA) [29, 30] and Random Forests (RF) [31, 32]. PCA is a method for reducing the dimensionality of the data in order to identify the parameters that explain the maximum variance in the data (Figs 2 and 3). In studies where the original variables are highly correlated, as is the case here, PCA can be used to reduce dimensionality without losing information and identify the most important variables. When the correlation between the original variables is weak, a larger number of components is necessary to capture the variability. The RF [31, 32] method, on the other hand, outputs the average of the results obtained from an ensemble of decision trees that it constructs by a bootstrapped sample from the original dataset (Fig 4). Here, a 'forest' of many trees is generated with the trees differing based on the composition of the training cases as well as the predictors used for each node. This method is considered highly robust due to the use of averaging and randomization. It also computes variable importance corresponding to the prediction.

A Random Forest is constructed by drawing a number of bootstrap samples (say, n) from the original sample. Then a classification tree is fit to each bootstrap sample resulting in n trees. The resulting set of trees is diverse since 1) the trees are unstable and change with learning data resulting in n trees that are different from each other, and 2) the splitting variables are randomly selected in each split resulting in a 'random forest' of trees. The RF algorithm produces two qualitative measures (Fig 4), namely, 1) the Increased Mean Square Error (IncMSE) and 2) the Increased Purity Index (IncNodePurity), that describe the predictive power of the original. The IncMSE is a measure of the predictive power when a value in the original is randomly permuted and if this random permutation changes the computed value significantly, then it is considered important. On the other hand, the IncNodePurity is a measure of the increase in homogeneity of a sample based on splitting them from a particular variable.

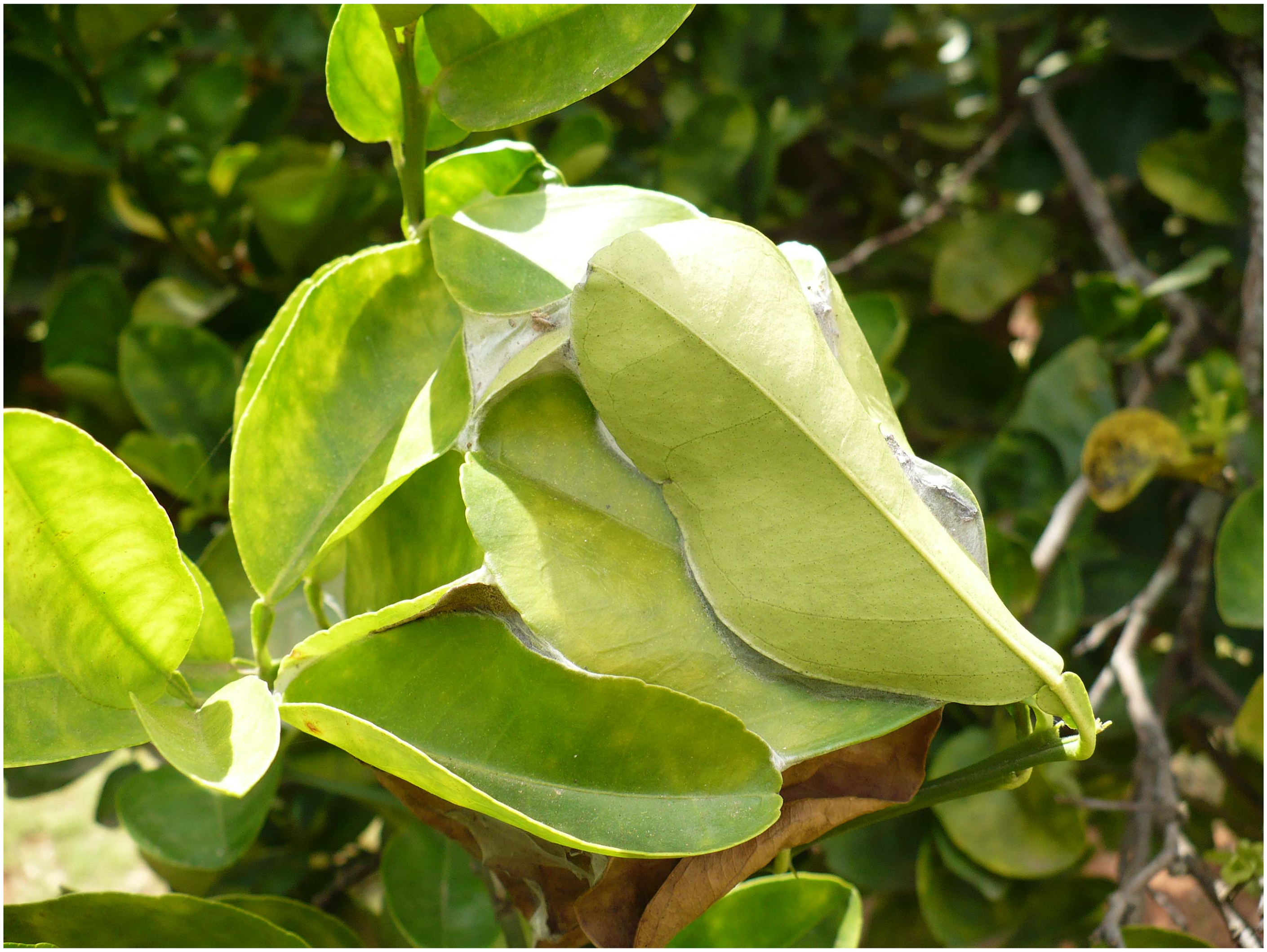

Fig 3. Biplot showing the principal componenents with the factors that contribute to explaining the most variation.

doi:10.1371/journal.pone.0156681.g003

We compared the linear PCA with the RF method for the same dataset. RF offers robust analysis as an alternative to the PCA and avoids the assumption of linearity between the new factors and the original variables required by PCA. The RF method is especially useful in the case of non-linear data that could have high-dimensionality [31, 32]. It is known to have high classification accuracy and an ability to model complex interactions among predictor variables [31, 32]. Moreover, it can deal with higher-order interactions as well as correlated predictor variables. However, this is biased in favor of continuous variables and variables with multiple categories.

The spatial arrangement was analyzed separately using visual exploration techniques (Figs 5 and 6). A 3-D visualization of each tree with the nests was made and the process automated such that visualizations can be made for any tree and any number of nests. The nests can be color-coded according to various parameters. In this case, the nests have been color-coded according to their nest size in terms of the number of leaves involved in their construction. This was implemented using the 3-D visualization toolkit Mayavi [33], using Python scripts.

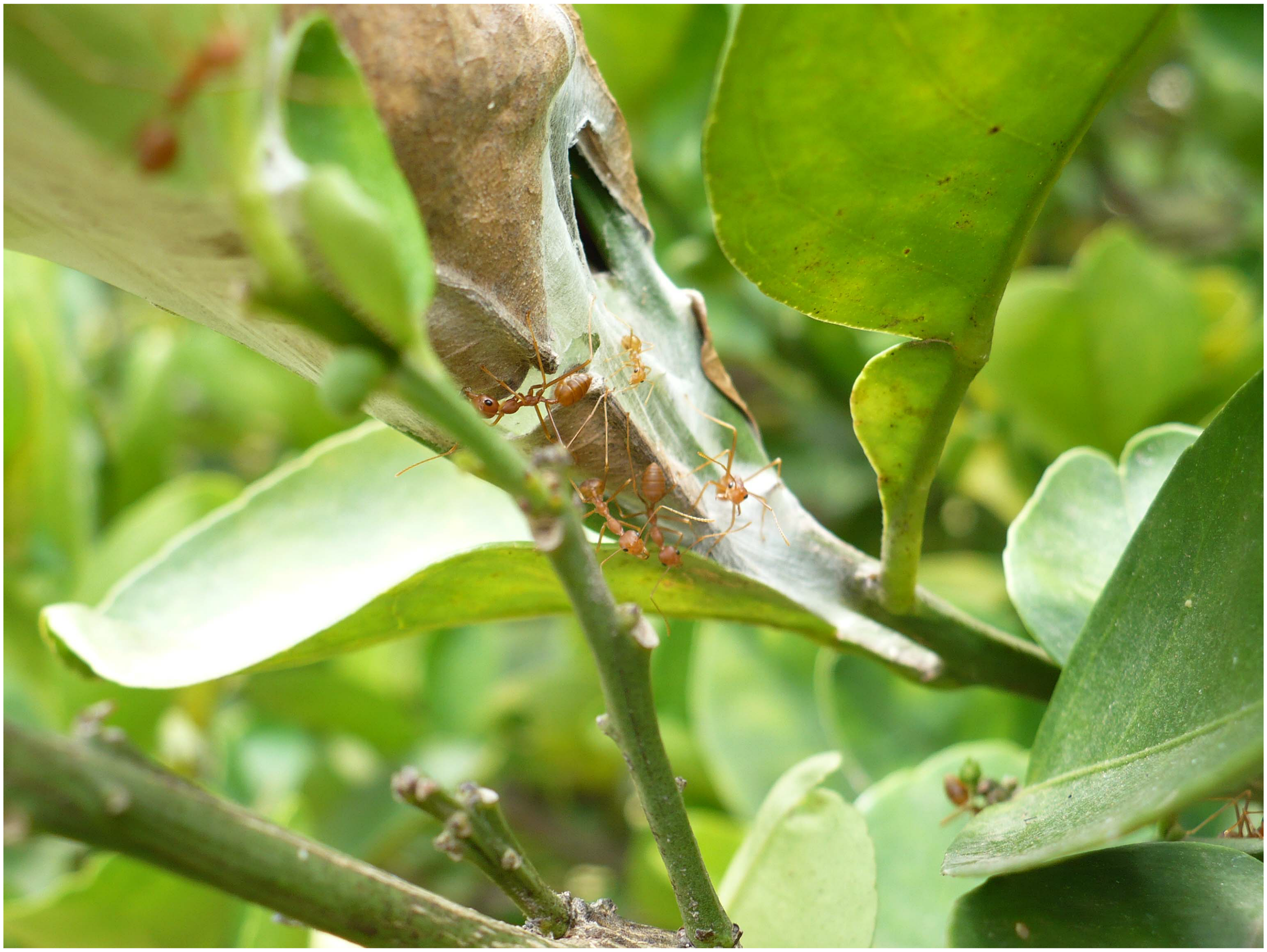

Fig 4. The VarImpPlot is a dotchart of variable importance as measured by a Random Forest. Here, the predictors are sorted in their increasing order of importance. TempOut: temperature outside the nest, CanopyRadius: canopy radius, Position: position on the tree, DisTrunk: distance of the nest from the trunk, TrunkHt: trunk height until location of first branching, NestSize: number of leaves used in construction of the nest, WindInt: wind intensity, TreeHt: total height of the tree, LeafLength: length of the leaf at the longest points, LeafBreadth: breadth of the leaf at the widest points, Luminosity: luminosity or ambient light, WindDir: direction of wind.

doi:10.1371/journal.pone.0156681.g004

Scientific visualization of the data was done to explore the attributes of tree morphology and nest characteristics to see if they could be predictors of the nest distribution in a tree.

The high-level model (Fig 5) of the tree involves approximation of the canopy to a hemisphere, the trunk to a cylinder, and nests as spheres, in addition to standard tree coloring. The geometric view of the tree is obtained by giving as input a file containing different parameters such as distance of the nest from the ground, distance of the nest from the tree trunk, and angular positions of the nests, and any tree with the requisite parameters can be simulated. Typically, trees will have branches jutting out unevenly and the shape of the canopy will be highly variable. Such branching has been ignored for the visualization resulting in nests being outside the actual canopy in the geometric view. This visualization of the trees is simply to understand the position of the nests and see if any patterns can be discerned by comparing across the different trees.

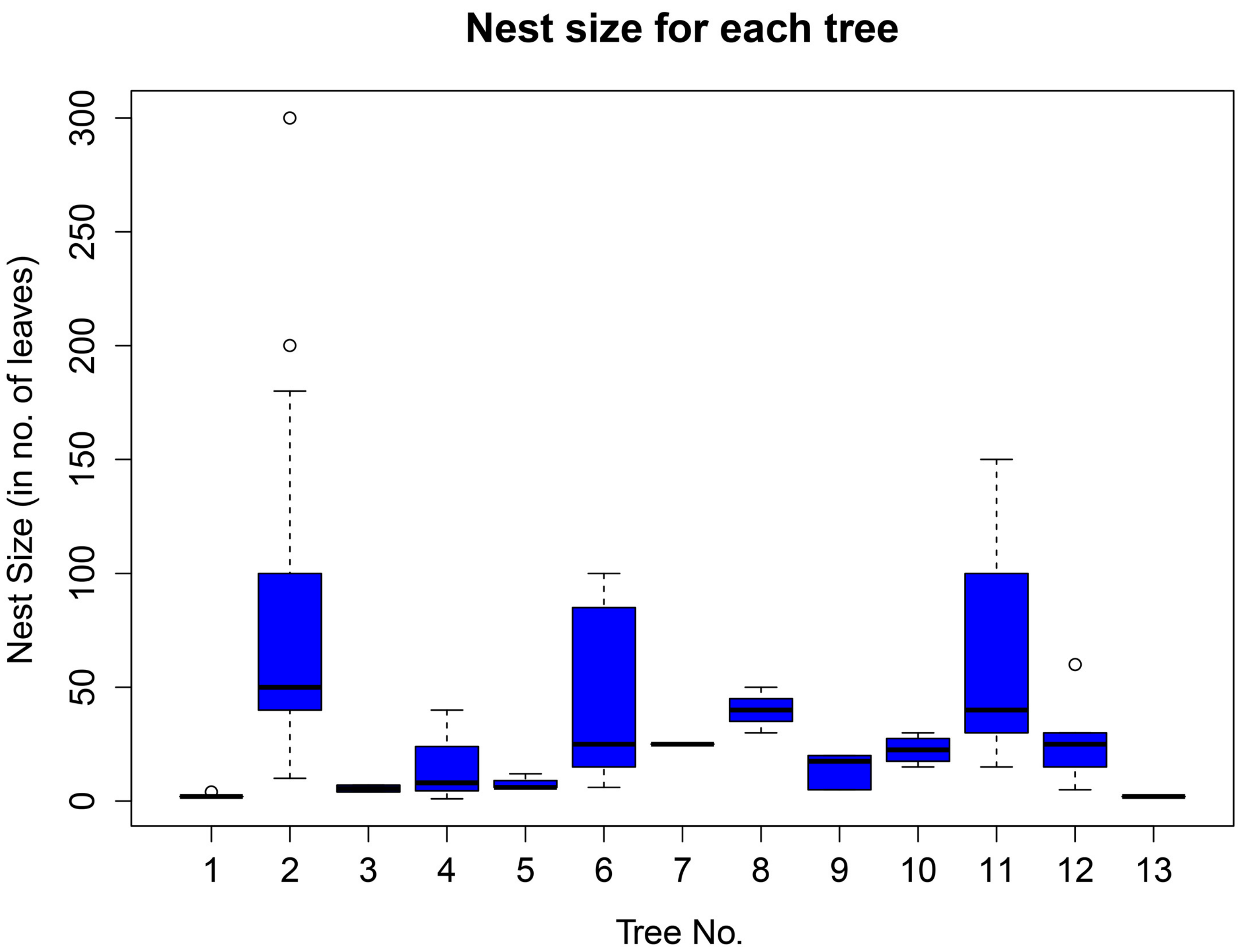

Fig 5. A 3-D model of tree no. 2 with with tree canopy approximated to a single hemisphere, and twenty weaver ant nests color-coded according to nest size in number of leaves. Some nests appear to be located outside the canopy since the hemisphere and the cylinder are just approximations of the actual tree and in reality the canopy is not a perfect hemisphere. Typically, there will be branches that are jutting out in different directions, which have been ignored in the visualization. Similarly, the nests are not spherical in reality and tend to be of different shapes and sizes.

doi:10.1371/journal.pone.0156681.g005

Both PCA and RF were implemented in the statistical package R version 2.15.2 (2012-10-26; The R Foundation for Statistical Computing). The RF analysis was performed using the 'randomForest' library. R was also used for all the exploratory data analyses. Python version 2.7.6 was used for the visualization along with the Mayavi [33] toolkit.

Results

Nest Structure and Construction

The workers of *Oecophylla* build nests by weaving together the leaves from the host plants using silk produced by their larva [1, 4, 5]. The workers typically manipulate the white larvae between their mandibles by touching each larva individually and one at a time onto target locations on the substrate. The points at which the larva touch the substrate (the leaf) result in the production of silk by the larva. This silky mesh exuded by the larva is used to bind the leaf

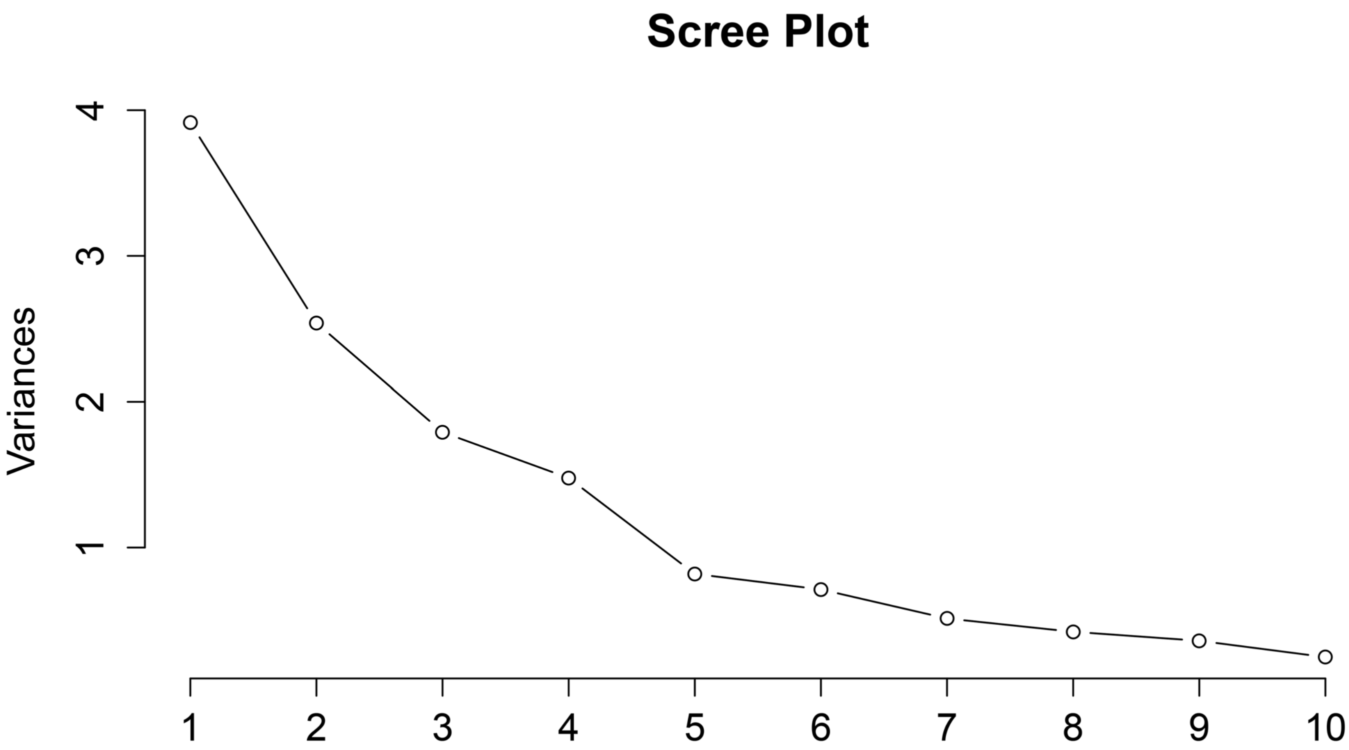

Fig 6. A panel of 3-D visualizations of 9 out of 13 trees oriented such that 90° out of the plane along the Y-axis (towards the reader) is the compass North. This is to compare the orientation of the nests which are positioned according to the absolute angles measured such that the Y-axis is the compass North. All nests have also been color-coded according to their size in terms of the approximate number of leaves used in construction.

doi:10.1371/journal.pone.0156681.g006

structures together (Fig 7). Occasionally, certain particles such as bark and soil are added to the larval silk mesh. The process was the same irrespective of whether 1) the weaving of nests was just starting, 2) the nests that are already built were extended, or 3) the existing nests were repaired.

A nest made from a single leaf was constructed by folding the leaf and stitching the leaf edges and tips together using the silk, which is typically white in color. Nests made of more leafs were stitched in a similar fashion, with leaves kept adjacent to each other such that their edges touched each other, but typically did not involve any folds in the leaves. In smaller trees with branches close to each other, the weaver ants used the available leaves on multiple branches close to each other such that some leaves on one branch were touching those on another, and stitched the leaves adjacent to each other. They did not require additional leaves to be brought for the sake of nest construction in such cases. There seemed to be very few chambers within the nest, if at all, from the nests observed. The really large nests were not accessible and hence the internal structure for these could not be discerned.

The nest structure was studied by dismantling eight abandoned nests and using six active nests from which the weaver ants were eliminated to the extent possible. Nest structure was also observed in nests as they were being constructed or repaired. The nests ranged in size from ones made with a single leaf to those constructed with tens or hundreds of leaves (Figs 8 and 9). The large ones, such as those made with leaves numbering roughly 60 or more, resembled bee-hives in shape and structure. These were frequently brown in color from the dry leaves unlike the green of the smaller and younger nests. In addition, the nest size of all 71 nests studied was recorded. Most of the trees had nests that were made up of fewer than 150 leaves and

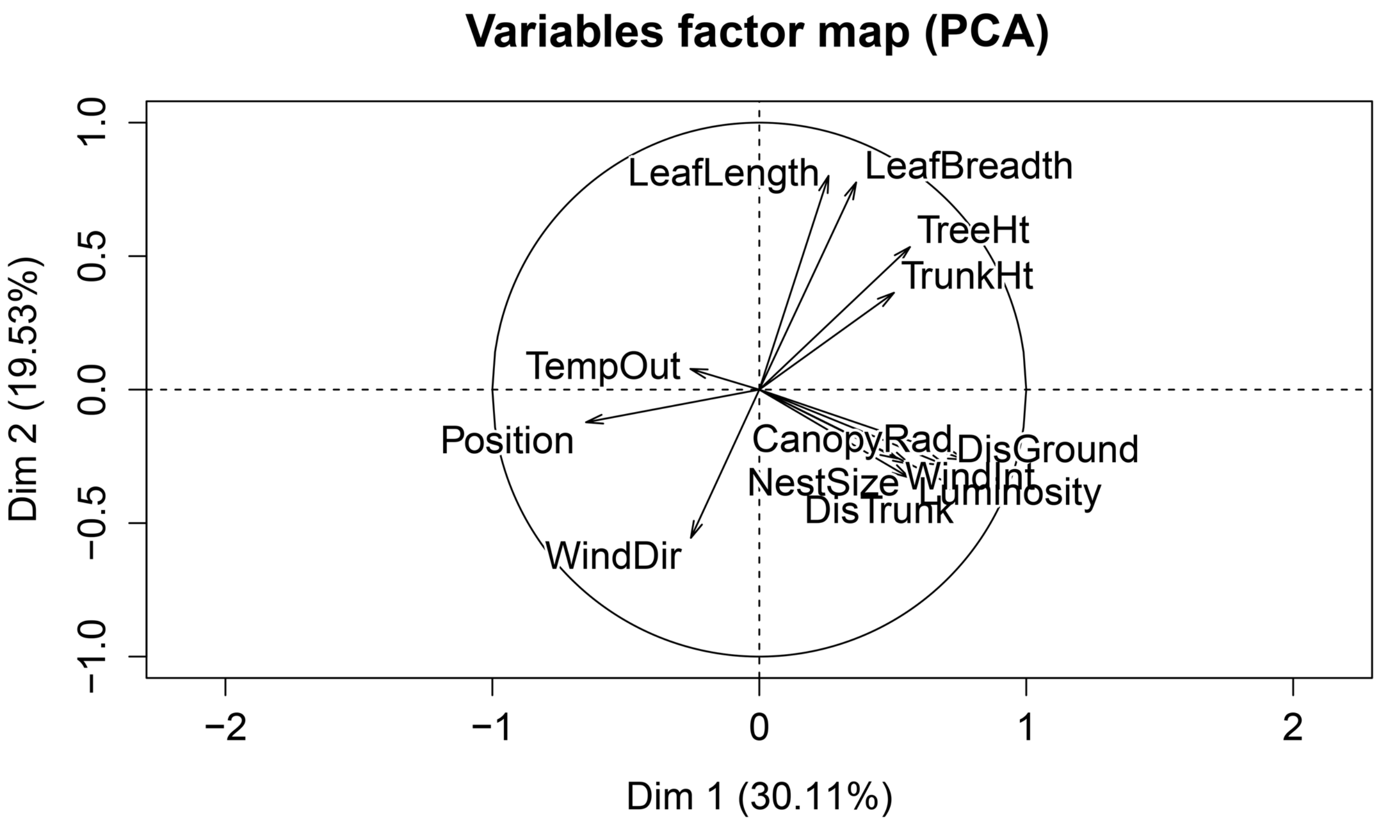

Fig 7. *Oecophylla smaragdina* constructing their nest on a lime tree.

doi:10.1371/journal.pone.0156681.g007

frequently involved fewer than 50 leaves in their construction (Fig 9).However, there were some nests which were quite large and comprised of more than 150 leaves, having up to a maximum of 300 leaves.

The nest size, in terms of the number of leaves used in the construction, emerged as an important factor in nest arrangement. Further, nest size seemed to be positively correlated with tree characteristics such as the canopy radius (Pearson $r^2 = 0.5$, $p<0.0001$). There was also a positive correlation between the position of the nest and the nest size. For instance, both the distance of the nest from the ground (Pearson $r^2 = 0.48$, $p<0.0001$) and the trunk of the tree (Pearson $r^2 = 0.53$, $p<0.0001$) seemed to correlate positively with nest size in number of leaves.

Nest Arrangement

Data on *O. smaragdina* nests were collected from 71 nests in 13 trees in both field sites. All the nests were found in fruit trees such as those belonging to the genera *Ficus*, *Citrus*, and *Azadirachta*. The PCA (Fig 2) showed that there were three principal components explaining 80% of the variation in the gathered data. The first principal component explained about 48% of the total variance and included the tree type and temperature outside the nest. The second principal component explained 18% and the third roughly 13%, leading to a cumulative of about 80%. The distance of the nest from the trunk, height of the nest, canopy radius and leaf size emerged as important factors (Fig 3). In general, the number of nests in trees with larger canopies were higher, and these also seemed to correspond with larger nests. Smaller trees typically had nests closer to the trunk than in the case of trees with larger canopies. This indicates that tree characteristics may be important determinants of nest arrangement in weaver ants. Unlike

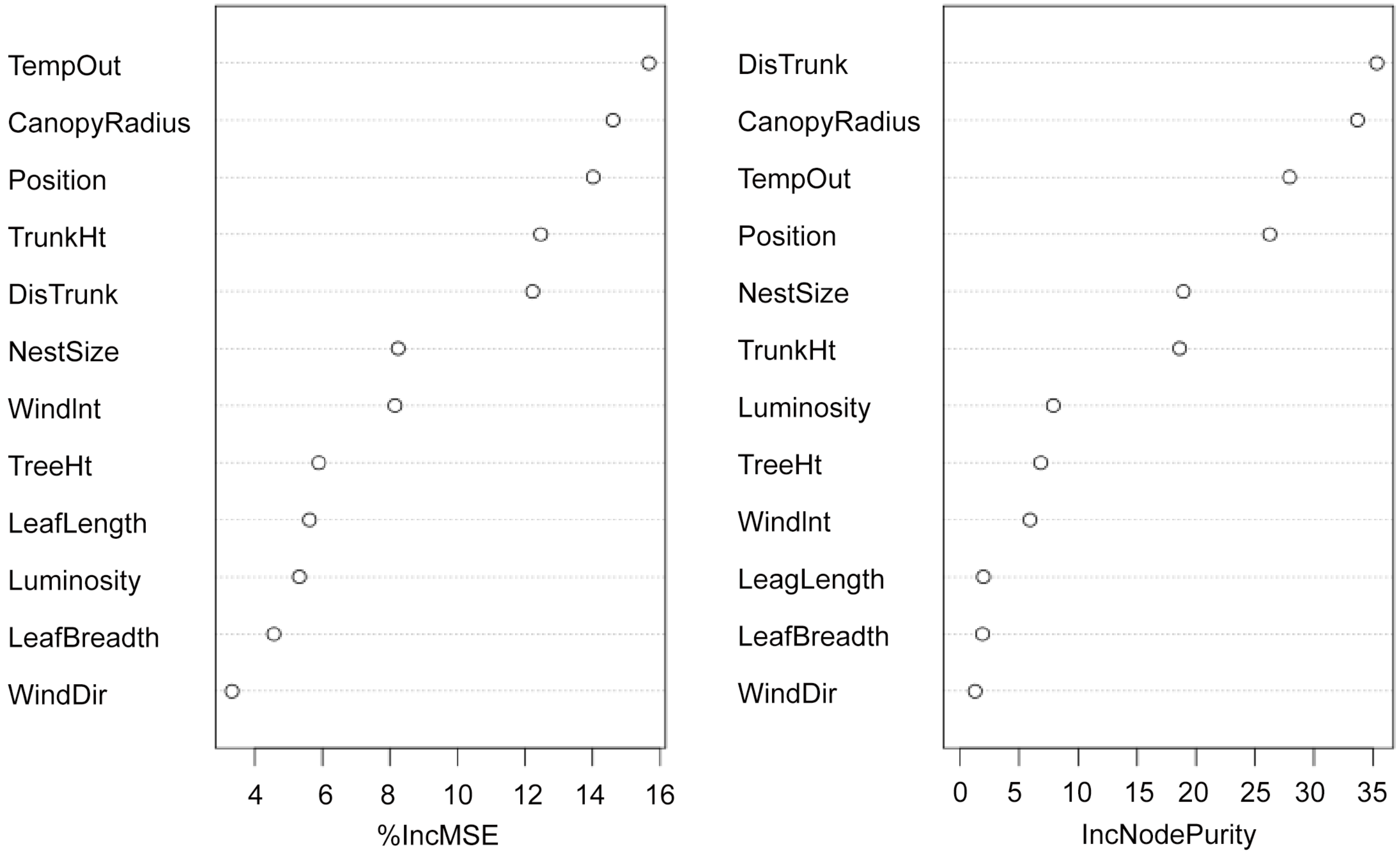

Fig 8. A typical Asian weaver ant nest.

doi:10.1371/journal.pone.0156681.g008

other colonial organisms, we think spatial constraints may not really be an important factor for weaver ant nests. However, access to resources could still be a crucial parameter in determining nest arrangement. To test this, we looked at individual characteristics of the variables used in PCA.

From the tree morphology, the canopy radius and the distance of nest from the trunk seemed to explain a large amount of the variance in the data, based on both PCA and RF methods (Fig 4). Leaf characteristics such as length and breadth emerged as important in PCA analyses but were not as significant in RF. Nest size (number of leaves used in the construction of the nest) seemed to be correlated to tree characteristics, as mentioned earlier. These indicate that tree and leaf characteristics seem to significantly influence the nest arrangement as well as nest architecture (Table 1). The best predictors of nest arrangement on the trees in the models constructed using RF were canopy radius, distance from the trunk, and temperature outside the nest (Fig 4). Abiotic factors such as wind intensity, wind direction, and luminosity did not seem to be as important as tree and leaf characteristics. Of the environmental variables, the temperature near the nest followed by wind intensity emerged as parameters explaining most of the variation. Consistently, even from exploratory data analysis, luminosity did not seem to be an important parameter.

The 3-D visualization was used to further understand how the different variables interacted with each other as well as influenced the arrangements of the nests in the individual trees. There was a clear preference for one side of the tree, consistent across the trees sampled, as well as a preference for certain heights (Mean = 3.2 m; SD = 1.7). In Fig 6, if out of the plane of the

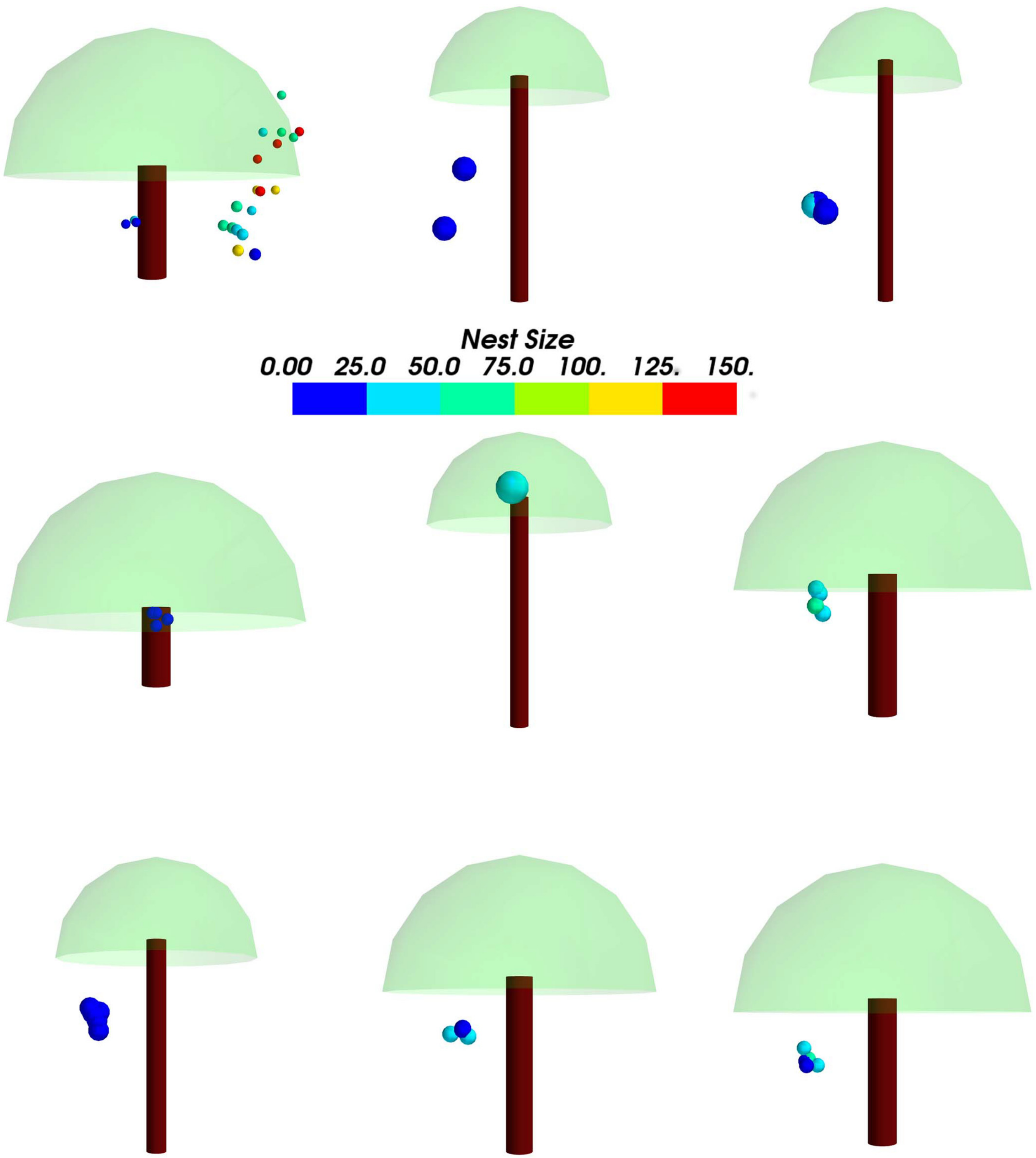

Fig 9. A boxplot of nest sizes in number of leaves for each of the 13 trees studied. A majority of the nests observed involved having less than 150 leaves used in their construction. However, some nests were made up of more than 150 leaves, having up to a maximum of 300 leaves.

doi:10.1371/journal.pone.0156681.g009

Table 1. A selection of original variables with their corresponding coefficient values based on PCA analyses of nest arrangement in Asian weaver ants.

Component	Variable	Coefficient
1	Distance from Tree Trunk	0.39
	Canopy Radius	0.39
	Distance from Ground	0.36
2	Leaf Length	0.50
	Leaf Breadth	0.49
	Wind Direction	0.35
	Tree Height	0.34

doi:10.1371/journal.pone.0156681.t001

image is the compass North, nests in eleven of the thirteen trees are clustered around the East. There could be a bias for height since some nests were too high to be measured. However, this bias was accounted for, since in all cases most nests on a given tree were measured, and only for one tree were some nests (three in number, out of more than twenty) ignored due to this.

Discussion

Given the lack of information about *Oecophylla smaragdina*'s nest distribution and arrangement, as well as the processes that determine resource selection in such invertebrates, the main aim of this study was to test some common hypotheses corresponding to weaver ants and habitat selection by them. This study has helped identify the important determinants of nest structure and arrangement, by showing that tree characteristics significantly influence choice of nest location in weaver ants. Since the leaves of the host plant are used in the construction of the nest by Asian weaver ants, we hypothesised that leaf characteristics such as size and shape could be important factors in nest location and arrangement. Our results from PCA seem to corroborate this hypothesis (Table 1). While certain environmental factors, such as temperature and wind intensity, too seem to affect the arrangement of weaver ant nests, these did not seem as significant as tree and leaf morphology. Our study also indicates that canopy size and tree height (referring to resource availability and tree architecture) are good predictors of nest arrangement in trees.

From the 3-D modeling (Fig 6), we could notice distributions clumped on certain parts of the tree, indicating a strong preference of certain micro-habitats over others. However, parameters such as wind direction and ambient light (which could be associated with this) were found to be inconsequential from the RF method. The temperature near the nest and wind intensity emerged as important among the environmental factors.

The process of determining the key factors that drive the selection of locations for nests is challenging and complex, especially in social insects such as weaver ants [6]. This is due to difficulties in demarcation of boundaries, since some colonies could be spread across multiple trees and large tracts of land. However, among invertebrates, ants are ideal subjects for studies involving habitat selection since they are highly mobile, often moving across micro-environments while foraging, and yet return to very specific habitats each time. This is especially true of weaver ants since they move across heterogenous habitats to their nests [1, 2, 21]. Moreover, unlike many other habitats of inverterbrates which can be difficult to find, these weaver ant nests are highly visible on the trees. Additionally, based on the dynamics over spatial and temporal scales, habitats could vary in terms of habitat quality. Due to these reasons, weaver ants proved to be ideal systems to study habitat selection in invertebrates [1, 2, 21].

Given such a context, the spatial arrangment of the nests can be highly informative about the types of biological and environmental pressures that can determine the life history of the organism. Thus, if one were to consider the habitat used as the realized niche and the habitat available as the potential niche, a crucial question is: why do weaver ants use certain micro-habitats and not others? Our results show that tree structure plays an important role in the size and position of nests on a tree, and some environmental variables could also be crucial to the arrangement of these nests.

Of the parameters considered, tree features such as the canopy radius, distance of the nest from the trunk, tree type, tree height and the position of the nest on the tree emerged as important factors, along with environmental factors such as the temperature near the nest. Leaf features such as size and shape were also shown to be factors that were crucial to nest arrangement. This helped understand how socially-living organisms interact with their environment and other organisms, and organize themselves in configurations that might protect them from factors detrimental to their fitness, such as predation-risk and proximity to resources.

Acknowledgments

I would like to thank Dr. Krushnamegh Kunte (NCBS) and Dr. Raghavendra Gadagkar (IISc) for their input and guidance. I would like to thank Dr. Sanjay Sane (NCBS) for permission to borrow a range of equipment crucial for the study at NCBS and Dr. Ajith Kumar (NCBS) for guidance and support. I thank Gaurav Agavekar and Ankan Chakraborty for field assistance, and Anupama Prakash for help in spotting nests at UAS. The sampling at IIT Madras would have been impossible but for Dr. Vinita Vasudevan (IITM) and Dr. Ramakrishna M. (IITM). I am indebted to Dr. Prabhu Ramachandran (IIT Bombay) for many insightful comments that helped improve this manuscript and assistance with Python. I am also grateful to two anonymous reviewers for comments that helped improve this manuscript. Lastly, I would like to thank NCBS for funding and logistical support.

Author Contributions

Conceived and designed the experiments: KD. Performed the experiments: KD. Analyzed the data: KD. Contributed reagents/materials/analysis tools: KD. Wrote the paper: KD.

References

1. Reh D. Ants that weave their own larvae. Scientific American Supplement. 1906; 1575:25241.
2. Peng R, Christian K, Gibb K. How many queens are there in mature colonies of the green ant. Australian Journal of Entomology. 1998; 37 (3):249–253. doi: 10.1111/j.1440-6055.1998.tb01579.x
3. Wilson E, Taylor R. A fossil ant colony: New evidence of social antiquity. Psyche. 1964; 71:93–103. doi: 10.1155/1964/17612
4. Holldobler B, Wilson E. Weaver Ants. Scientific American. 1977; 237.
5. Cole A, Jones J. A Study of the weaver ant *Oecophylla smaragdina*. American Midland Naturalist. 1948; 39 (3):641–651. doi: 10.2307/2421529
6. Schluns E, Wegener B, Schluns H, Azuma N, Robson S, Crozier R. Breeding system, colony and population structure in the weaver ant *Oecophylla smaragdina*. Molecular Ecology. 2008; 18:156–167. doi: 10.1111/j.1365-294X.2008.04020.x PMID: 19076274
7. Lach L, Hoffman B. Are invasive ants better plant-defense mutualists? A comparison of foliage patrolling and herbivory in sites with invasive yellow crazy ants and native weaver ants. Oikos. 2010; 120 (1):9–16. doi: 10.1111/j.1600-0706.2010.18803.x
8. Bluthgen N, Fiedler K. Interactions between weaver ants *Oecophylla smaragdina*, homopterans, trees and lianas in an Australian rain forest canopy. Journal of Animal Ecology. 2002; 71:793–801. doi: 10.1046/j.1365-2656.2002.00647.x

9. Mele PV. A historical review of research on the weaver ant *Oecophylla* in biological control. Agricultural and Forest Entomology. 2008; 10:13–22.
10. Way M. An insect pest of coconuts and its relationship to certain ant species. Nature. 1951; 168:302. doi: 10.1038/168302b0 PMID: 14875079
11. Peng R, Christian K, Reilly D. The effect of weaver ants *Oecophylla smaragdina* on the shoot borer *Hypsipyla robusta* on African mahoganies in Australia. Agricultural and Forest Entomology. 2011; 13:165–171. doi: 10.1111/j.1461-9563.2010.00514.x
12. Sribandit W, Wiwatwitaya D, Suksard S, Offenberg J. The importance of weaver ant (*Oecophylla smaragdina Fabricius*) harvest to a local community in Northeastern Thailand. Asian Myrmecology. 2008; 2:129–138.
13. Mele PV, Camara K, Vayssieres J. Thieves, bats and fruitflies: Local knowledge on the weaver ant *Oecophylla smaragdina* in relation to three 'invisible' intruders in orchards in Guinea. International Journal of Pest Management. 2008; 55 (1):57–61. doi: 10.1080/09670870802450276
14. Holldobler B, Wilson E. Colony-specific territorial pheromone in the African weaver ant *Oecophylla longinoda* (*Latreille*). Proceedings of the National Academy of Sciences. 1977; 74 (5):2072–2075. doi: 10.1073/pnas.74.5.2072
15. Bradshaw J, Baker R, Howse P. Multicomponent alarm pheromones of the weaver ant. Nature. 1975; 258:230. doi: 10.1038/258230a0 PMID: 1238905
16. Jander R, Jander U. The light and magnetic compass of the weaver ant *Oecophylla smaragdina* (*Hymenoptera*: *Formicidae*). Ethology. 1998; 104:743–758. doi: 10.1111/j.1439-0310.1998.tb00108.x
17. Dlussky G, Wappler T, Wedmann S. New middle Eocene formicid species from Germany and the evolution of weaver ants. Acta Palaeontologica Polonica. 2008; 4:615–626. doi: 10.4202/app.2008.0406
18. Santos J, Yamamoto M, Oliveira F, Del-Claro K. Behavioral Repertory of the Weaver Ant *Camponotus* (*Myrmobrachys*) *senex* (*Hymenoptera*: *Formicidae*). Sociobiology. 2005; 45 (1).
19. Jaisson P. Environmental preference induced experimentally in ants (*Hymenoptera*: *Formicidae*). Nature. 1980; 286:388. doi: 10.1038/286388a0
20. Newey P, Robson S, Crozier R. Weaver ants *Oecophylla smaragdina* encounter nasty neighbours rather than dear enemies. Ecology. 2010; 91 (8):2366–2372. doi: 10.1890/09-0561.1 PMID: 20836458
21. Bochynek T, Robson SKA. Physical and biological determinants of collective behavioural dynamics in complex systems: Pulling chain formation in the nest-weaving ant *Oecophylla smaragdina*. PLoS ONE. 2014; 9 (4)(4). doi: 10.1371/journal.pone.0095112 PMID: 24759886
22. Beattie A. The evolutionary ecology of ant-plant mutualisms. Cambridge University Press; 1985.
23. Dunn R, Gove A, Barraclough T, Givnish T, Majer J. Convergent evolution of an ant-plant mutualism across plant families, continents, and time. Evolutionary Ecology Research. 2007; 9:1349–1362.
24. Edwards D, Hassall M, Sutherland W, Yu D. Selection for protection in an ant-plant mutualism: Host sanctions, host modularity, and the principal-agent game. Proceedings of the Royal Society B. 2006; 273 (1586):595–602. doi: 10.1098/rspb.2005.3273 PMID: 16537131
25. Janzen D. Coevolution of mutualism between ants and *Acacias* in Central America. Evolution. 1966; 20 (3):249–275. doi: 10.2307/2406628
26. Palmer T. Spatial habitat heterogeneity influences competition and coexistence in an African *Acacia* ant guild. Ecology. 2003; 84:2843–2855. doi: 10.1890/02-0528
27. Stanton M, Palmer T, Young T, Evans A, Turner M. Sterilization and canopy modification of a swollen thorn *Acacia* tree by a plant-ant. Nature. 1999; 401:578–581. doi: 10.1038/44119
28. Stanton M, Palmer T, Young T. Competition-colonization trade-offs in a guild of African *Acacia*-ants. Ecological Monographs. 2002; 72:347–363. doi: 10.1890/0012-9615(2002)072%5B0347:CCTOIA%5D2.0.CO;2
29. Hotelling H. Analysis of a complex of statistical variables into principal components. Journal of Educational Psychology. 1933; 24:498–520. doi: 10.1037/h0070888
30. Hill M, Smith AJE. Principal component analysis of taxonomic data with multistate discrete characters. Taxon. 1976; 25:249–255. doi: 10.2307/1219449
31. Breiman L. Random forests. Machine Learning. 2001; 45:5–32. doi: 10.1023/A:1010933404324
32. Cutler DR, Edwards TC, Beard KH, Cutler A, Hess KT, Gibbs JC, et al. Random forests for classification in ecology. Ecology. 2007; 88:2783–2792. doi: 10.1890/07-0539.1 PMID: 18051647
33. Ramachandran P, Varoquaux G. Mayavi: 3D visualization of scientific data. IEEE Computing in Science and Engineering. 2011; 13 (2):40–51. doi: 10.1109/MCSE.2011.35
